# The Impact of *Piriformospora indica* on plant heat and drought tolerance

**DOI:** 10.3389/fpls.2024.1479561

**Published:** 2024-12-06

**Authors:** Hao Ji, Min Zhang, Chuanhuang Huang, Wei Lin, Yin Lu, Peijie Wang, Bin Dong, Bizhu He, Binghua Wu, Lijin Guo

**Affiliations:** ^1^ International Magnesium Institute, College of Resources and Environment, Fujian Agriculture and Forestry University, Fuzhou, China; ^2^ College of Life Sciences, Fujian Agriculture and Forestry University, Fuzhou, China; ^3^ Fuzhou Botanical Garden, Fuzhou, China; ^4^ College of Forestry, Fujian Agriculture and Forestry University, Fuzhou, China; ^5^ International College, Fujian Agriculture and Forestry University, Fuzhou, China; ^6^ College of Bee Science and Biomedicine, Fujian Agriculture and Forestry University, Fuzhou, China; ^7^ College of Horticulture, Fujian Agriculture and Forestry University, Fuzhou, China

**Keywords:** drought stress, *Piriformospora indica*, stress resistance, high-temperature stress, climatic change

## Abstract

In recent years, the global rise in temperatures has led to drought and heat becoming major environmental stresses that limit plant growth. Previous research has demonstrated the potential of *Piriformospora indica* in augmenting plant stress resistance. However, specific studies on its effects and underlying mechanisms in cuttings of *Rosa chinensis, Jasminum sambac*, and *Rhododendron simsii* Planch are relatively limited. The objective of this study is to explore the effects and mechanisms of *P. indica* on cuttings and tissue-cultured seedlings of these plants under conditions of drought and high-temperature stress. The experiment involved subjecting *P. indica*-inoculated and non-inoculated plants to drought (one week without watering) and high-temperature (24-hour exposure to 45°C) stress in a controlled environment chamber. Indicators such as chlorophyll content, chlorophyll fluorescence parameter Fv/Fm, and antioxidant enzyme activity were measured. The results showed that inoculation with *P. indica* significantly increased the survival rate of the three types of plant cuttings under drought conditions by 13%, 17%, and 16.6% respectively, and resulted in a substantial decrease in malondialdehyde content alongside an increase in chlorophyll content. Under high-temperature stress at 45°C, the chlorophyll fluorescence parameter Fv/Fm values increased by 27.3%, 10.3%, and 51.1% compared to the control group. Furthermore, heat tolerance tests at 42°C showed a 2% higher survival rate in the *P. indica* inoculated *Rhododendron* tissue-cultured seedlings than in the control group, with a positive effect observed on the activities of superoxide dismutase and peroxidase. These findings demonstrate that inoculation with *P. indica* significantly enhances the resistance of *Rhododendron, Jasminum sambac*, and *Rosa* to drought and high-temperature stresses, providing insights for sustainable agricultural development and the comprehensive exploitation of the potential value of *P. indica*.

## 1 Introduction

*Piriformospora indica* (*P. indica*) is known to significantly enhance plant stress resistance, yet its underlying mechanisms remain to be clarified (Verma et al., 1998). Discovered within the roots of shrubs in the Thar Desert of northwestern India, *P. indica* is a microbe that exhibits potent growth-promoting effects. It not only effectively improves the nutrient absorption capacity of host plants and stimulates growth but also strengthens systemic resistance against a variety of environmental threats (Saddique et al., 2018). With the exacerbation of the greenhouse effect leading to a global rise in temperatures. Over the past decade, IPCC reports have highlighted a significant surge in the frequency and intensity of events such as flooding and high temperatures. This observed trend, contrary to previous assumptions, suggests a probable escalation in the future (Lee et al., 2023). Drought and high temperature are projected to be the primary limiting factors for plant growth in the future (Moss et al., 2010). Several studies have indicated that inoculation with *P. indica* markedly improves plant resistance to drought, salinity, and disease (Boorboori and Zhang, 2022; Mensah et al., 2020; Paul et al., 2023; ur Rahman et al., 2023). These effects are attributed to *P.indica*’s promotion of a series of biochemical reactions within plants, including but not limited to the enhancement of antioxidant enzyme activities (Boorboori and Zhang, 2022), accumulation of osmoprotectants (Chen et al., 2022), and regulation of plant hormone levels (Leyva-Rojas et al., 2020). Additionally, *P.indica* may also enhance the plant’s perception and response to environmental stress by activating signaling pathways (Li et al., 2022, 2023). While contemporary studies have unveiled the prospective role of *P. indica* in augmenting plant stress resilience, a more comprehensive examination of its specific mechanisms is imperative. Consequently, delving deeper into the correlation between *P. indica* inoculation and heightened plant stress resilience will not only enrich our comprehension of plant-microbe interactions but also furnish novel avenues for mitigating climate change impacts and fostering sustainable agricultural advancement.

Research indicates that inoculation with *P. indica* can significantly enhance a plant’s drought resistance. While the precise mechanisms of action have not been fully revealed, current studies have explored several potential mechanisms. A widely accepted mechanism is that *P. indica* modulates plant hormone levels, such as increasing concentrations of gibberellin (GA) and indole-3-acetic acid (IAA) gibberellin (GA) and indole-3-acetic acid (IAA) levels, to promote root development and enhance water absorption capacity, thereby improving drought resistance (Aruna et al., 2023; Jisha et al., 2019; Mohsenifard et al., 2017). At the same time, some scholars have pointed out that Indian pear-shaped spores can also improve the drought resistance of plants by affecting the defense hormones in plants. Jogawa et al. showed that *P. indica* inoculation can enhance plant tolerance to abiotic stress by regulating the signaling pathways of plant defense hormones such as JA and SA. The article particularly emphasized the key role of these hormones in plant defense responses and stress tolerance (Jogawat et al., 2013). Waller et al. proposed that *P.indica* improves barley tolerance to stress (including drought) by upregulating the expression of salicylic acid (SA)-related genes and inducing disease resistance-related signaling pathways (Waller et al., 2005). Furthermore, *P. indica* may increase the content of osmoprotective substances (such as proline and sugars) within the plant, helping to maintain cellular osmotic balance and mitigate water loss under drought stress (Rajput et al., 2022). Baltruschat et al. found that *P. indica* can enhance barley’s tolerance to abiotic stresses (including drought) by increasing the content of osmotic regulating substances such as proline and soluble sugars (Baltruschat et al., 2008). Sun et al. found that *P. indica*. can enhance the drought resistance of Chinese cabbage by changing proline and carbohydrate metabolism and regulating ABA signal transduction (Sun et al., 2010). Additionally, *P. indica* is known to activate stress response genes, inducing the expression of stress-related genes such as drought-responsive element-binding proteinsheat and shock proteins (HSPs), thereby enhancing plant adaptation to drought stress (Mohsenifard et al., 2017). Some scholars have also suggested that *P. indica*’s potential mechanisms in improving plant drought tolerance may include enhancing the activity of antioxidant enzymes within the plant, reducing the damage caused by reactive oxygen species (ROS) to plant cells, and thus protecting plants at the cellular level from drought stress (Kundu and Vadassery, 2022; Tyagi et al., 2017). Despite existing research providing insights into how *P. indica* improves plant drought tolerance, some gaps remain. For instance, most studies focus on observing phenotypic changes after *P. indica* inoculation, with insufficient research on the underlying molecular mechanisms. Moreover, comparative studies on the effectiveness of *P. indica* in enhancing drought resistance across different plant species are relatively lacking, limiting our comprehensive understanding of its scope and effects. In summary, future research needs to delve deeper into the specific pathways of these mechanisms and expand to more plant species and various environmental conditions to fully assess the capacity for *P. indica* in raising crop drought resistance in actual agricultural production (Aruna et al., 2023; Jisha et al., 2019; Mohsenifard et al., 2017). *P. indica* has been found to have a certain impact on improving plant heat tolerance, but research on its role in enhancing plant heat resistance is relatively limited, and its mechanisms have not been fully elucidated (Rajput et al., 2022; Kundu and Vadassery, 2022; Tyagi et al., 2017). Preliminary studies suggest that *P. indica* may enhance plant heat tolerance through various mechanisms. Firstly, it promotes the activity of the plant’s antioxidant enzyme system, alleviating oxidative stress caused by high temperature (Li et al., 2023; Wu et al., 2022). Secondly, it may regulate plant hormone levels, improving plant growth conditions and the ability to cope with high temperature (Gill et al., 2016; Kumar Bhuyan et al., 2015). Lastly, it activates the expression of heat stress-related genes, improving plant cell tolerance to thermal stress (Ghaffari et al., 2019; Hosseini et al., 2018; Xu et al., 2017). Moreover, existing research primarily focuses on small-scale trials under laboratory conditions, lacking large-scale validation in field conditions. The specific molecular mechanisms and physiological processes by which *P. indica* affects plant heat tolerance still require further investigation. Therefore, future research should not only delve into the specific mechanisms of *P.indica* in enhancing plant heat tolerance at the molecular and physiological levels but also pay attention to its effects across different crop species, growth stages, and environmental conditions. This could lead to the development of more effective biotechnological strategies to enhance crop adaptation to increasingly severe high-temperature stress.

To understand more precisely and systematically the function of *P. indica* in improving drought and heat tolerance in plants., this study selected cuttings of *Rhododendron*, *Jasminum sambac*, and roses, as well as tissue-cultured *Rhododendron* seedlings as research subjects. We aim to comprehensively assess the impact of *P. indica* inoculation on cellular enzyme activity, chlorophyll content, and malondialdehyde (MDA) levels in these plants under drought conditions. Simultaneously, the effect of *P. indica* inoculation on cuttings’ chlorophyll fluorescence parameters and *Rhododendron* tissue-cultured seedlings’ cellular enzyme activity and survival under high temperature is investigated. Based on preliminary observations and literature reviews, we propose three hypotheses: (1) *P. indica* may enhance plant stress resistance by optimizing cellular membrane permeability and boosting the activity of chlorophyll synthesis-related enzymes. (2) *P. indica*, which activates key anti-oxidant enzymes such as superoxide dismutase (SOD) and peroxidase (POD), may be a primary mechanism for improving plant drought and heat tolerance. (3) *P. indica* may also regulate the efficiency of light energy conversion in photosystem II (PSII) and affect the generation of reactive oxygen species, further modulating plant adaptation to high temperatures. This study aims to elucidate the mechanisms by which *P. indica* promotes plant stress resilience, providing new strategies for agricultural production in the face of extreme climatic conditions and promoting the sustainable development of agricultural ecosystems.

## 2 Experimental materials and methods

### 2.1 Plant materials

The plant specimens utilized in this study were *Rosa chinensis*, *Rhododendron*, and *Jasminum sambac*, all sourced from the campus gardens of Fujian Agriculture and Forestry University (119°23′41″ E, 26°08′75″ N). These specimens were subsequently subjected to cutting and tissue culture experiments within the controlled environment of our laboratory. Both inoculated and non-inoculated specimens with *P. indica* were prepared for the inoculation experiments.

### 2.2 Handling of cuttings

Cuttings of *Rosa chinensis*, *Rhododendron*, and *Jasminum sambac* were collected from carefully selected mother plants on the campus. For *Rosa chinensis* cuttings, healthy shoots from the current year’s growth were chosen. The upper part was cut flat, while the lower part was cut at a 45-60 degree angle using a sharp knife or pruning shears. For *Jasminum sambac* cuttings, new shoots from the current year with at least two pairs of buds were selected. Cuts were made close to the nodes, approximately 1 cm below a node, with all side branches and leaves from the middle and lower parts removed. For *Rhododendron* cuttings, robust branches were selected, and the lower leaves were removed, leaving only 2-3 leaves at the top.

After preparing all the cuttings, two treatments-CK and Pi-were applied to the materials. First, all cuttings were soaked in a 1:1000 carbendazim solution for 1 hour and then thoroughly rinsed with tap water to prevent contamination by various pathogens before applying the different treatments. CK Treatment, Insert the pretreated cuttings directly into the substrate and water with tap water every 3 days.Pi Treatment, The pre-treated cuttings were directly inserted into the substrate, and 2 g of fungal blocks were mixed into every liter of the substrate. For the first month, 6 g L^-1^ of fungal solution was used to water the cuttings every three days, for a total of 10 applications.The cultivation substrate used for all treatments consisted of a 1:1 mixture of perlite and vermiculite. The substrate was sterilized in advance with high-pressure steam at 121°C for 20 minutes and then filled into 50-cell trays. All experiments were conducted in the artificial climate chamber at the College of Horticulture, Fujian Agriculture and Forestry University. The photoperiod was set to 16 hours light/8 hours dark, with day/night temperatures at 26°C/20°C and humidity at 80%. During the first month, appropriate dark treatments were applied. Each treatment included at least 15 cuttings and was replicated more than three times.

### 2.3 Preparation and inoculation of tissue culture seedling medium

#### 2.3.1 Preparation of the Culture Medium

The main components are sucrose at 30 g L^-1^ and agar at 4.5-5.0 g L^-1^, with the pH adjusted to 5.6. Preparation of the Culture Medium: the main components are sucrose at 30 g L-1 and agar at 4.5-5.0 g L-1, with the pH adjusted to 5.6. After preparation, the medium is sterilized in an autoclave at 121°C for 20 minutes. It is then cooled to room temperature and kept ready for use. The specific components of the Rhododendron bud differentiation induction medium are listed in **Table 1**. (1) Freshly harvested leaves are stored at -4°C for 12-24 hours. (2) Before tissue culture, the leaves are rinsed with tap water for 30 minutes. (3) The leaves are disinfected with 75% alcohol for 30 seconds. (4) The leaves are sterilized with either mercuric chloride for 5-8 minutes or Clorox (bleach) solution for 10-15 minutes. (5) After cutting the leaves into sections, they are soaked in sterile water or liquid medium for 10 minutes. (6) The leaves are then placed on filter paper to dry. (7) Finally, the leaves are placed in culture bottles.

**Table 1:** Components of *rhododendron* bud differentiation induction medium.

Basal medium	Plant growth regulator (mg L^-1)^	
read	IAA	ZT
	10	10

#### 2.3.2 Culture Conditions

*Rhododendron* contains high levels of phenolic compounds, which can lead to oxidation during tissue culture. Therefore, after completing the tissue culture procedures, the explants are incubated in the dark at 4°C for 2 days. They are then transferred to fresh medium and cultured in the dark at 25°C for 2-3 weeks. After the dark incubation, the explants are cultured under low light conditions (1500 lux, 16/8h light/dark cycle) at 25°C in the tissue culture room. The medium is replaced every month. Depending on the growth stage of the tissue culture materials, they are sequentially transferred to callus induction, shoot bud induction, and rooting induction media to complete the entire growth process.

Inoculation of *P. indica:* Using a hole punch, create a hole in the center of a freshly prepared PDA medium plate. Place a small *P. indica* plug, with the mycelium side facing down, into the hole created by the punch. Seal the edge of the petri dish with parafilm and incubate it in the dark at 28°C.

After one week of culturing, *P. indica* can be used for inoculating *Rhododendron* tissue culture seedlings. For inoculation, use a sterile 200 μL pipette tip to cut a 5 mm diameter fungal plug from the edge of the *P. indica* colony. Place the plug approximately 1 cm away from the root of a tissue culture seedling that has been growing on rooting medium for one week. The seedlings are then cultured for an additional two weeks.

### 2.4 Trypan blue staining detection

After one month of different treatments on the cuttings, staining identification was performed on the *Piriformospora indica*-inoculated seedlings.

1. Preparation of Staining Solution: Weigh 0.81 g of sodium chloride, 0.06 g of disodium hydrogen phosphate, and 0.5 g of Trypan Blue powder, and dissolve them in 60 ml of ultrapure water. After thorough dissolution, make up the volume to 100 ml and store the solution at room temperature (Türkkan, 2015)
2. Staining Procedure: Select the upper part of the roots, clean off the soil, and cut them into 1 cm pieces. Place the root segments in 10% KOH and incubate in a boiling water bath for 1 hour. After cooling to room temperature, rinse with distilled water and soak overnight. Then, immerse in 1% HCl for 10 minutes, followed by staining with 0.05% Trypan Blue for 6-8 hours. Rinse with distilled water 3-5 times, soak in sterile water for 1 hour, and then prepare slides for observation under a light microscope.

### 2.5 Solid culture of *P. indica*

After washing and peeling, 200 g of potatoes were weighed and chopped into small pieces. They were then added to 800 mL of distilled water and boiled until fully cooked. The mixture was filtered multiple times through four layers of gauze. Then, 20 g of dextrose and 20 g of agar were added to the filtrate, and distilled water was added to make up the volume to 1 L. The solution was boiled to dissolve, aliquoted into Erlenmeyer flasks, and sterilized in an autoclave at 121°C for 20 minutes. The specific formulation of the PDA solid and PDB liquid media for *P. indica* spore culture is shown in **Table 2**. After cooling slightly, the solution was poured into petri dishes and sealed for later use, forming Potato Dextrose Agar (PDA) medium.

**Table 2:** *P. indica* spore culture medium formula.

Components	PDA solid medium	PDB liquid medium
Potato	200g	200g
Glucos	20g	20g
Agar	20g	
ddH_2_O	Dilute to 1L	Dilute to 1L

#### 2.5.1 Liquid Culture of *P. indica*

The Potato Dextrose Broth (PDB) medium has the same formula as PDA, except without agar. Using a sterile 200 μL pipette tip, 2-3 fungal plugs with a 5 mm diameter were cut from the edge of a 7-day-old *P. indica* culture grown on PDA. These plugs were placed in the PDB medium and cultured on a shaker (28°C, 200 rpm) for 3 days.

#### 2.5.2 Reactivation of *P. indica* Strains

Using a sterile 200 μL pipette tip, a 3 mm diameter fungal plug was cut from the edge of the colony and inoculated onto PDA solid medium plates. The plates were incubated at 28°C in the dark for 7 days, after which they were ready for use.

#### 2.5.3 Preparation of *P. indica* Spore Suspension

The experimental *P. indica* strains were obtained from the laboratory. The activated fungal plugs were inoculated into PDA liquid medium and cultured on a shaker at 28°C and 200 rpm for 3 days to obtain a mycelium rich in spores. The mycelium was filtered multiple times through four layers of gauze, After sufficient growth, 6 grams of fungal mycelium and spores were harvested and suspended in 1 liter of sterile distilled water to create the fungal solution.ground, and prepared into a *P. indica* spore suspension at a concentration of 6 g L ^-1^for later use (Deshmukh et al., 2006).

### 2.6 High-temperature stress experiment

*Rhododendron* tissue-cultured seedlings and cuttings, both inoculated and non-inoculated with *P. indica*, were placed in a BIC-400 artificial climate chamber (Boxun, Shanghai, China) under a constant light intensity of 12,000 lux, a 16 h/8 h light cycle, and relative humidity maintained at 80%. The treatment temperatures were set at 42°C for tissue-cultured seedlings and 45°C for cuttings, with a duration of 24 hours. Following treatment, leaves from similarly grown seedlings and cuttings under different conditions were selected for staining experiments. Determination of the maximum quantum efficiency Fv/Fm of chlorophyll fluorescence parameters PSII of the leaves of the cutting seedlings with three replicates for each treatment.

### 2.7 Drought stress experiment

Cuttings, both inoculated and non-inoculated with *P. indica*, were divided into two groups and subjected to drought stress experiments in an artificial climate room at a temperature of 25°C, light intensity of 10,000 lux, and a 16 h/8 h (day/night) light cycle. The drought stress was induced by withholding water for a week, with leaf sampling conducted on days 0, 3, and 7. The MDA content was determined using the thiobarbituric acid method. Staining experiments were also conducted post-drought stress, with each parameter measured in triplicate to minimize experimental variability. The final data were averaged and presented with standard deviations.

### 2.8 DAB and NBT staining

To detect reactive oxygen species in plants subjected to high temperature and drought treatments, seedlings were stained using DAB (3,3’-diaminobenzidine) and NBT (nitro blue tetrazolium) (Thordal-Christensen et al., 1997). The seedlings were first thoroughly washed with distilled water to remove any surface impurities. They were then placed in 50 ml tubes, fully submerged in a freshly prepared DAB or NBT staining solution, and incubated overnight at room temperature in the dark to prevent photooxidation. After incubation, the staining solution was carefully replaced with anhydrous ethanol. The plant material was then boiled in a water bath with anhydrous ethanol, replenishing the ethanol as necessary, until complete decolorization was achieved, leaving only the stained areas visible. Following decolorization, the stained plant tissues were photographed using a root scanner to capture the results.

### 2.9 Data processing

Data collection and analysis of the experimental results were performed using Microsoft Excel. The experiment was conducted with three biological replicates, each consisting of a mixed sample of three plants, and each sample was technically replicated three times to minimize experimental randomness. The data were averaged and displayed with standard deviations using GraphPad Prism 8.0. Statistical analysis to find significant differences between means was performed using ANOVA (Analysis of Variance), followed by Tukey’s HSD (Honestly Significant Difference) test for multiple group comparisons.

## 3 Results

### 3.1 Observation of the colonization of Piriformospora indica in the roots of results of trypan blue staining

**Figure 1:**
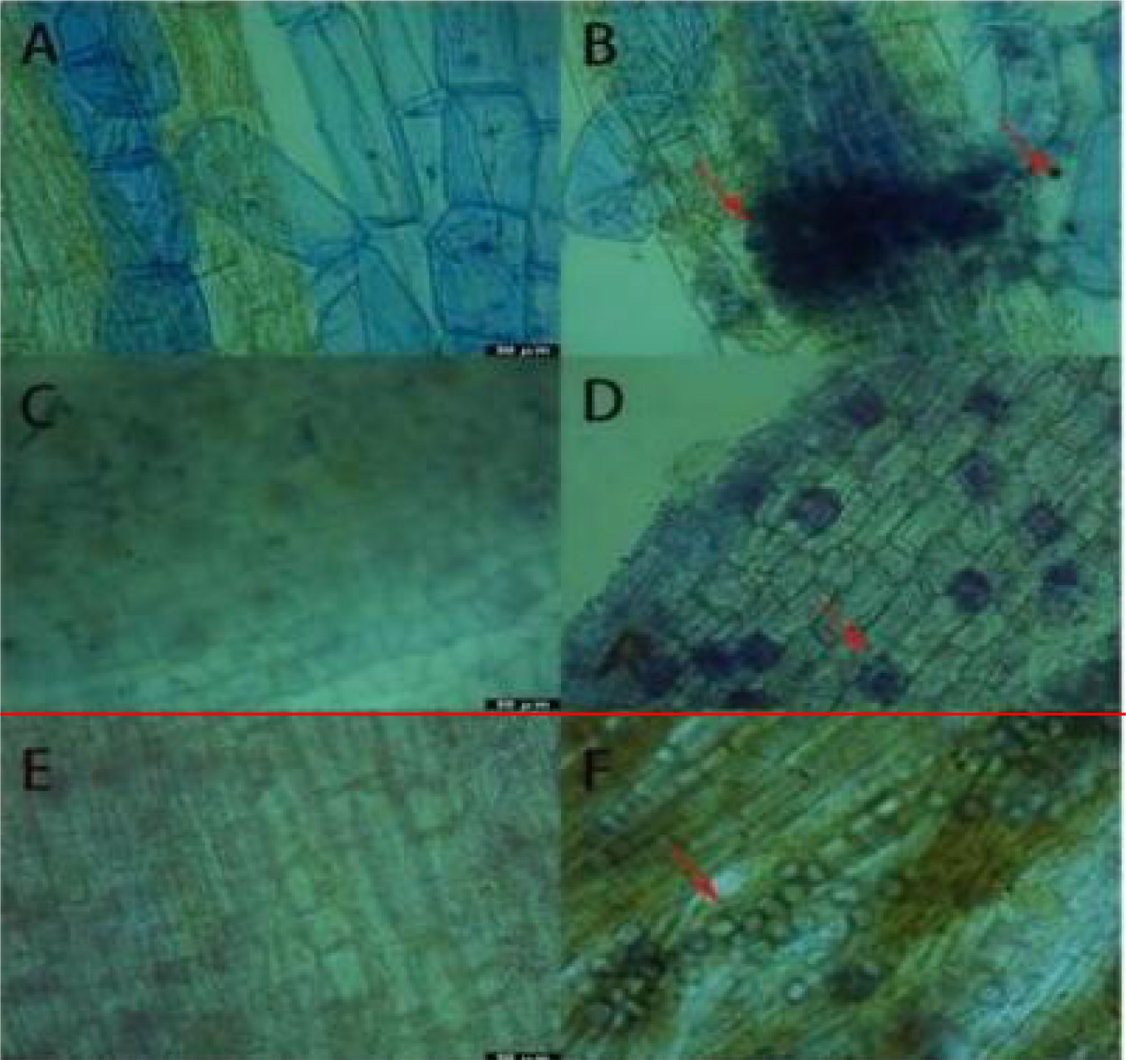
Results of Trypan Blue Dyeing. **(A)** Root system of wild-type *Rhododendron*. **(B)** Root system of *Rhododendron simsii* inoculated with *Pi*. **(C)** Root system of wild-type *Jasminum sambac*. **(D)** Root system of *Jasminum sambac* inoculated with *Pi*. **(E)** Root system of wild-type *Rosa chinensis* cuttings. **(F)** Root system of *Rosa chinensis* inoculated with *Pi* Arrow: Spores.

Results (**Figure 1**) indicate that following inoculation with *P. indica*, successful colonization of spores on the roots of cuttings was clearly observed under an optical microscope using trypan blue staining. In *Rhododendron* roots, both spores and mycelia were visible, with a notable aggregation of mycelia and spores surrounding them, though the mycelia were not fully dispersed. In the root systems of *Jasminum sambac* and *Rosa chinensis*, characteristic pear-shaped, thick-walled spores were primarily observed around root hair and epidermal cells, with few present in the central cylinder of the roots. The spatial distribution of spores within the different root systems appeared random. In *Rosa chinensis*, spores were often arranged in chains, whereas in *Jasminum sambac*, they were more dispersed, which may be attributed to differences in root system architecture. Notably, *Rosa chinensis* and *Jasminum sambac* roots exhibited a higher density of spore colonization compared to *Rhododendron*.

### 3.2 Effect of *P. indica* on wilting severity of cuttings under drought stress

**Figure 2:**
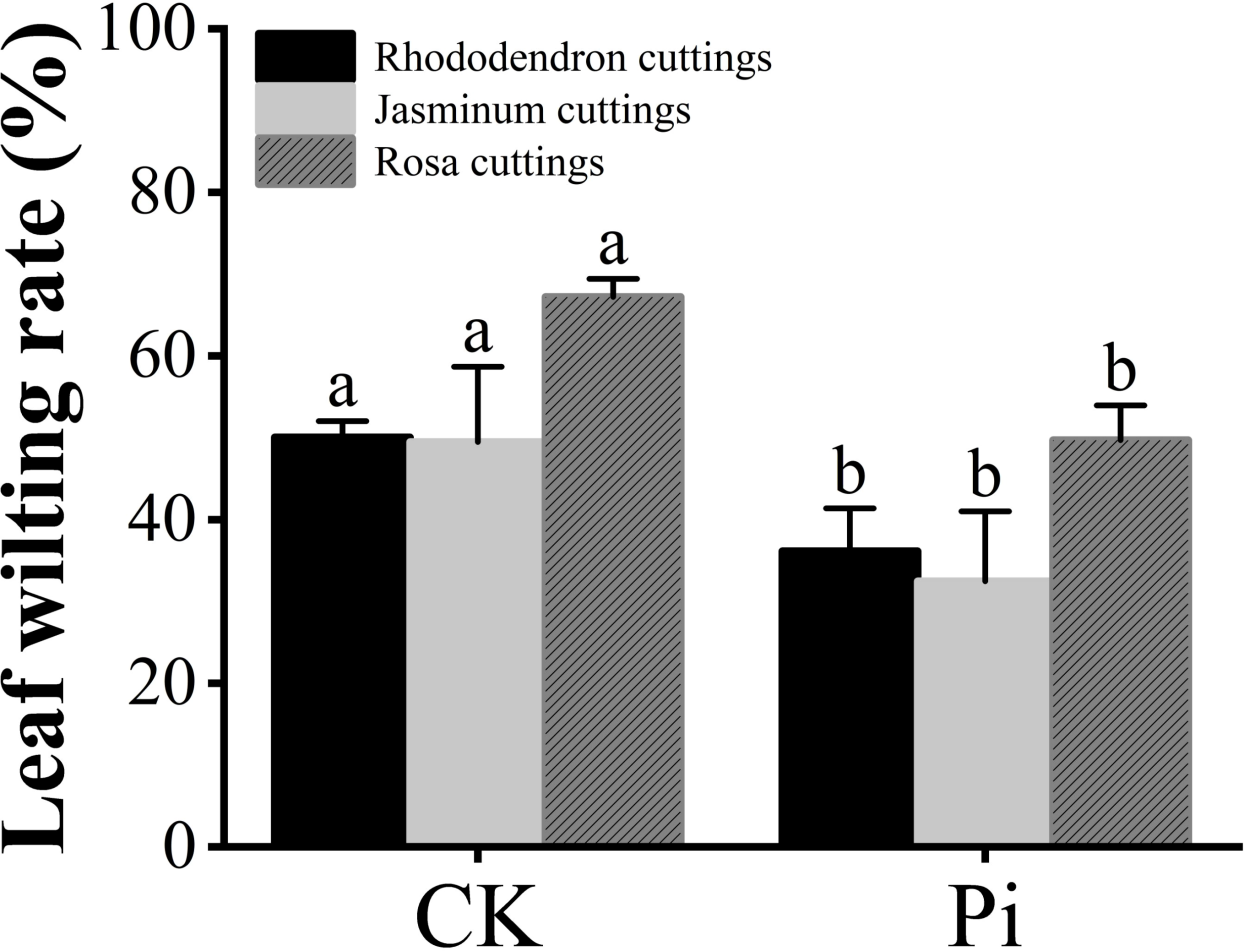
Statistics of leaf wilting rate before and after drought stress. The small letters (such as 'a' and 'b') in the bar graphs of your figures typically indicate statistical significance among the treatments, which is often determined through post-hoc analysis after ANOVA testing, such as Tukey's test or LSD (Least Significant Difference) test.

**Figure 3:**
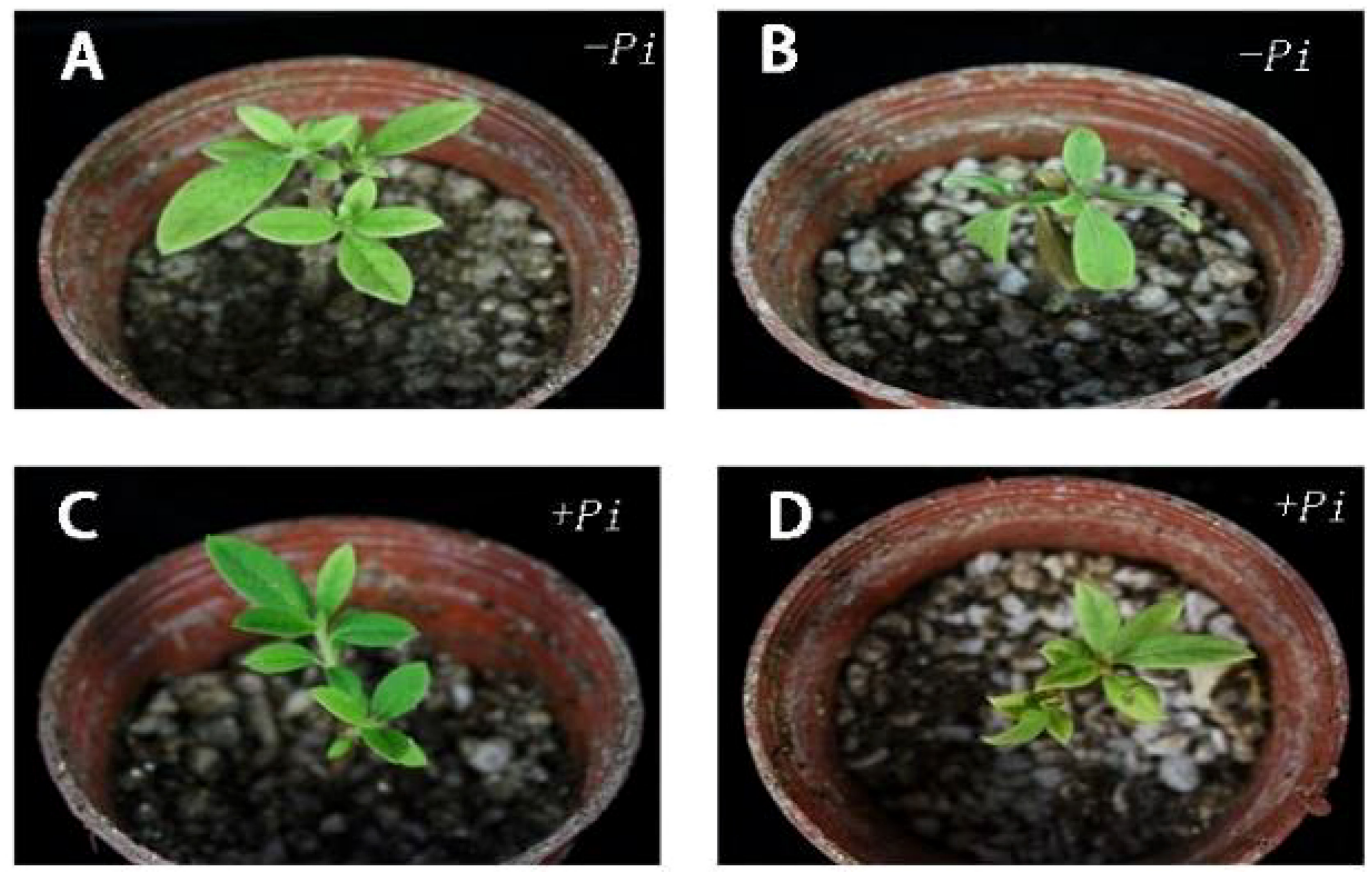
Phenotypic difference of *Rhododendron* cuttings before and after drought stress and leaf wilting rate. **(A)** Before drought stress of cuttings inoculated with *Piriformospora indica*: **(B)** After drought stress of cuttings inoculated with *Piriformospora indica* for 7 days: **(C)** Before the drought stress of the cuttings inoculated with *Piriformospora indica* 7 days: **(D)** After the drought stress of the cuttings inoculated with *Piriformospora indica* for 7 days.

For the drought tolerance experiment, three cuttings each of *Rhododendron*, *Jasminum sambac*, and *Rosa chinens* is were selected for different treatments. One week post drought stress, the phenotypes of both *P. indica-inoculated (Pi)* and non-inoculated plants were observed. The leaf wilting rate was quantified (**Figures 2**, **3**), with *Pi*-inoculated cuttings exhibiting a lower degree of wilting compared to the control. The experimental results revealed that out of 30 leaves in the *Rhododendron* control group, 15 exhibited wilting, whereas in the *P. indica* group, 13 out of 35 leaves wilted, resulting in wilting rates of 50% and 37%, respectively.

**Figure 4:**
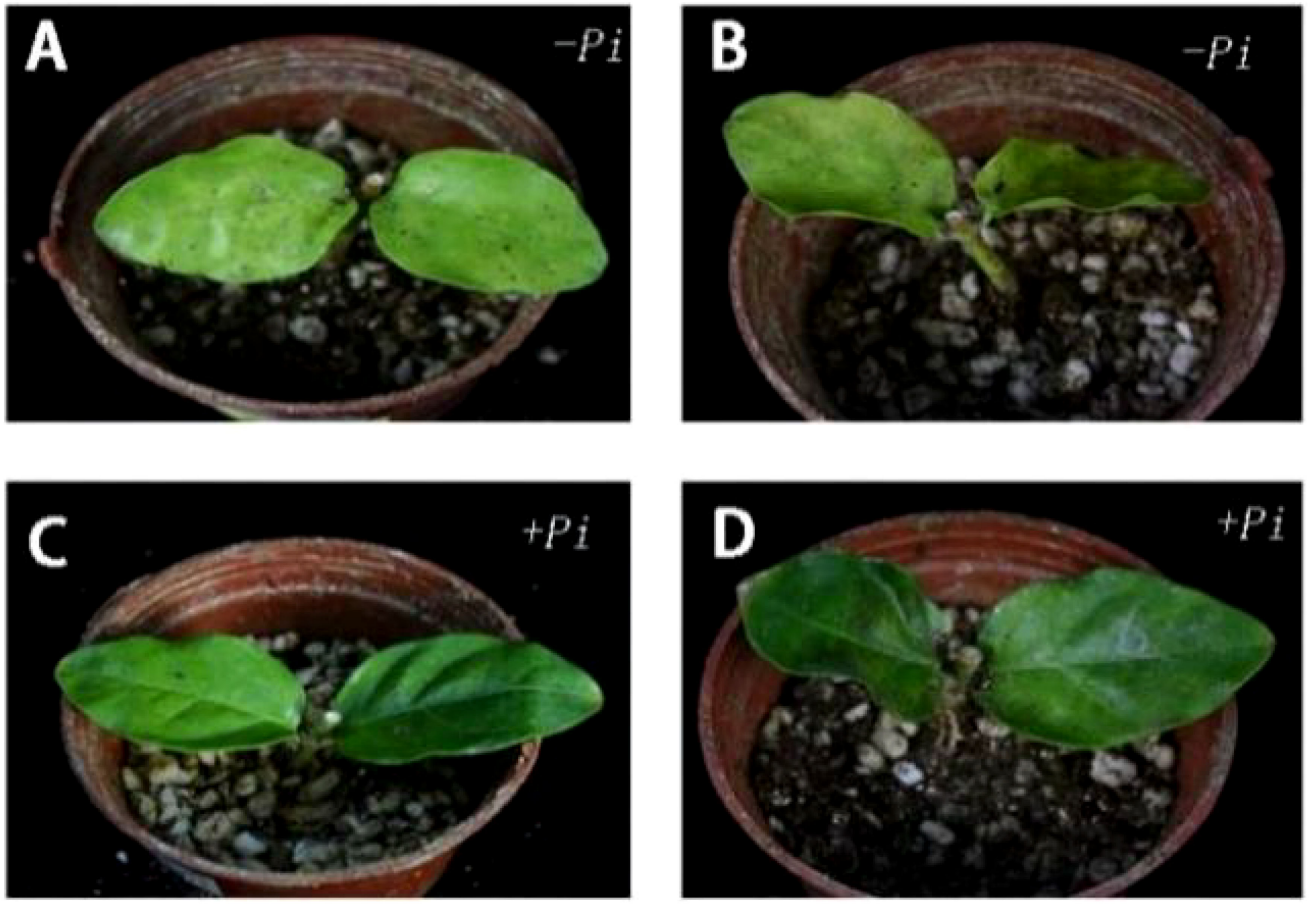
Phenotypic difference of *Jasminum sambac* cuttings before and after drought stress and leaf wilting rate. **(A)** Before drought stress of cuttings inoculated with *Piriformospora indica:* **(B)** After drought stress of cuttings inoculated with *Piriformospora indica* for 7 days: **(C)** Before the drought stress of the cuttings inoculated with *Piriformospora indica* 7 days: **(D)** After the drought stress of the cuttings inoculated with *Piriformospora indica* for 7 days.

Experimental results, as shown in **Figures 2** and **4**, indicate that for the *Jasminum sambac* control group (CK), out of 6 leaves, 3 exhibited wilting, whereas in the *P. indica*-inoculated (*Pi*) group, out of 6 leaves, 2 exhibited wilting, resulting in wilting rates of 50% and 33%, respectively. Phenotypic observations showed that the degree of leaf curling in the *Jasminum sambac* plants inoculated with *Pi* was considerably reduced compared to that in the controls.

**Figure 5:**
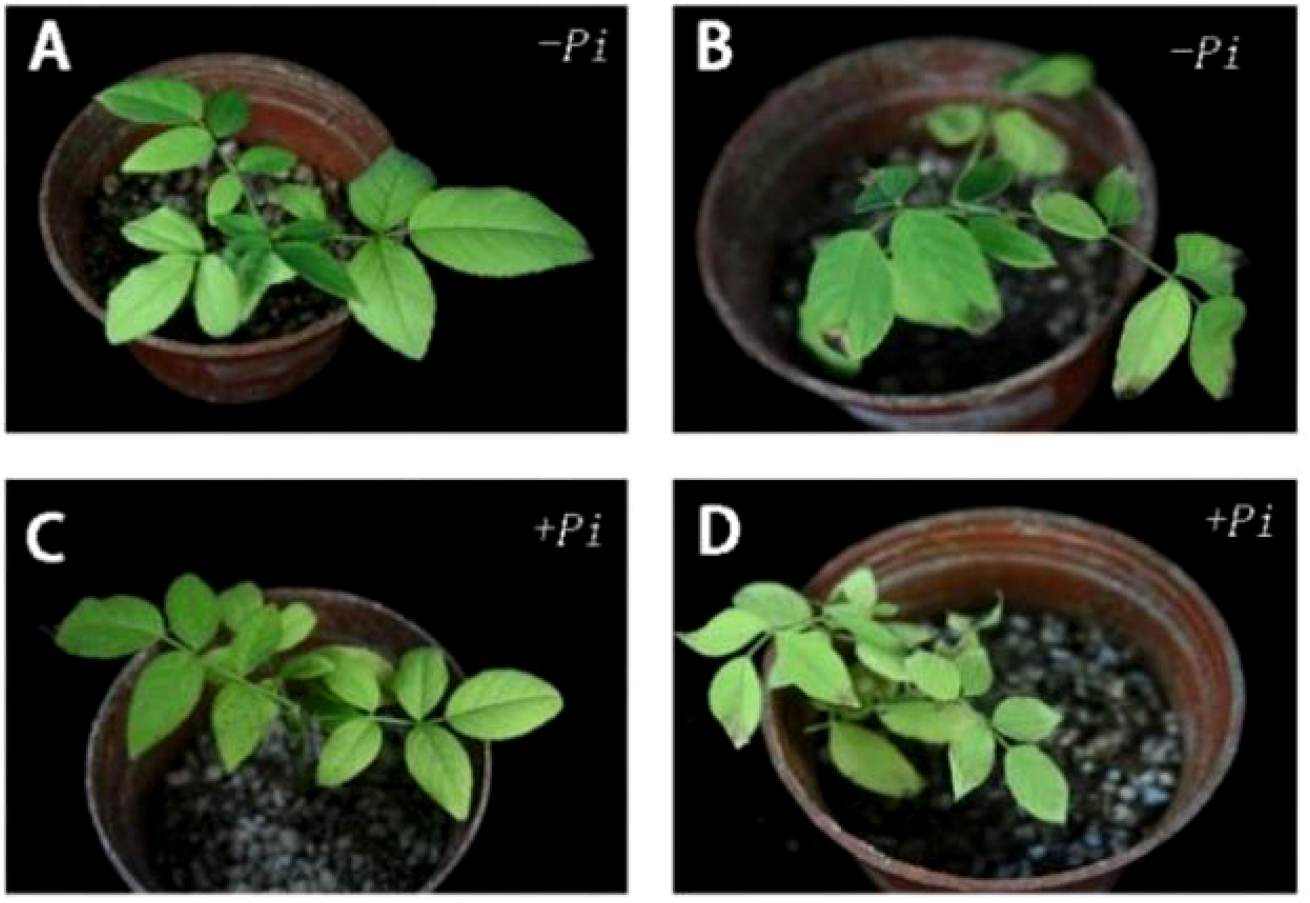
Phenotypic difference of *Rosa chinensis* cuttings before and after drought stress and leaf wilting rate. **(A)** Before drought stress of cuttings inoculated with *Piriformospora indica*. **(B)** After drought stress of cuttings inoculated with *Piriformospora indica* for 7 days. **(C)** Before the drought stress of the cuttings inoculated with *Piriformospora indica* 7 days. **(D)** After the drought stress of the cuttings inoculated with *Piriformospora indica* for 7 days.

Wilting rate statistics for *Rosa chinens is* leaves, as depicted in **Figure 2**, show that out of 45 leaves in the *Rosa chinensis* CK group, 30 exhibited wilting, while in the *Pi* group, out of 40 leaves, 20 exhibited wilting, with wilting rates of 66.6% and 50%, respectively. Phenotypic observations showed that wilting severity was reduced in the *Pi*-inoculated *Rosa chinensis* compared to the controls, and the control showed leaf tip browning in **Figure 5**.

### 3.3 The impact of *P. indica* on malondialdehyde content in cuttings under drought stress

**Figure 6:**
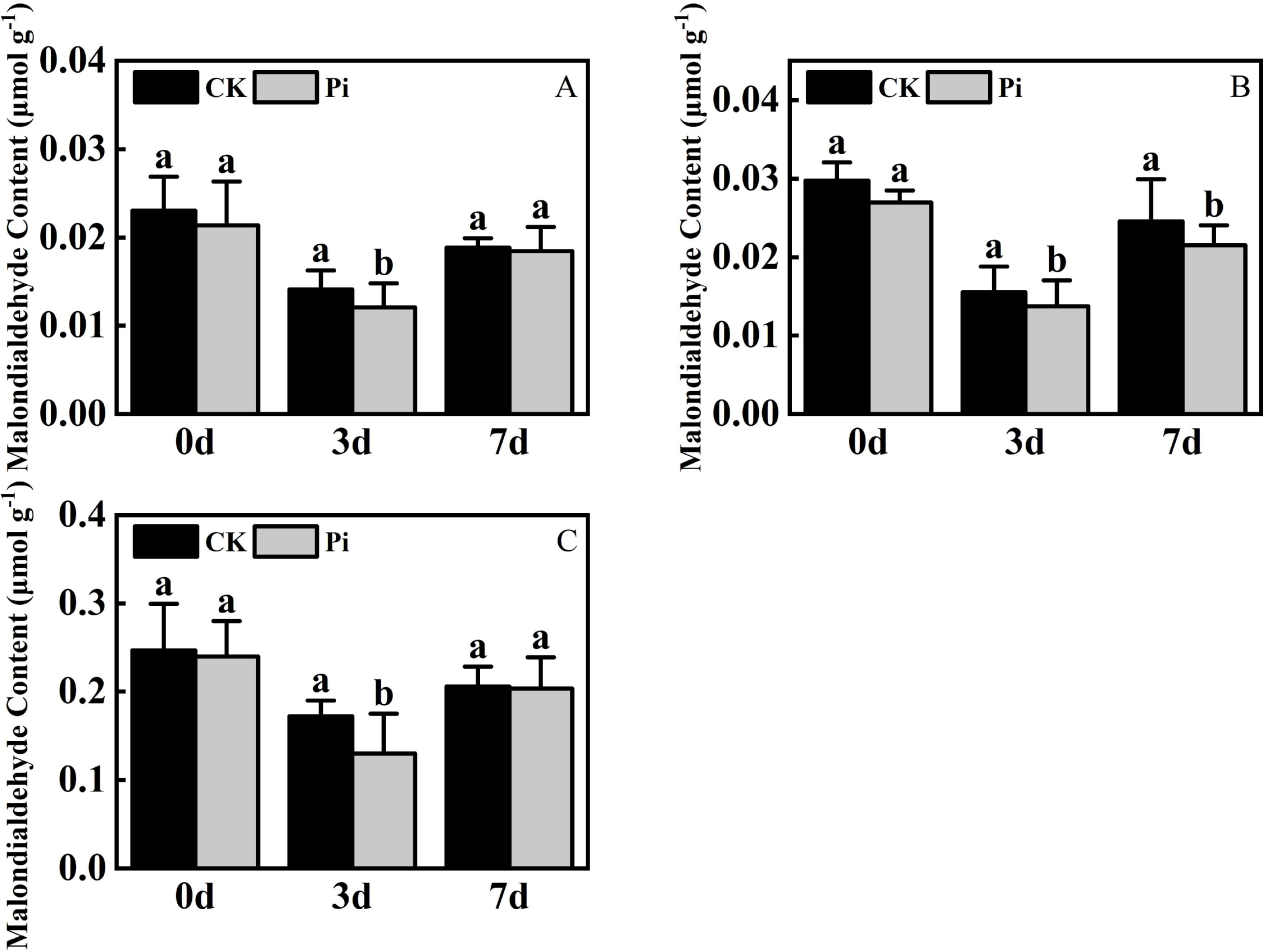
Malondialdehyde Content after drought stress. **(A)** Malondialdehyde content in *Rhododendron* seedlings after drought stress; **(B)** Malondialdehyde content in *Jasminum sambac* seedlings after drought stress; **(C)** Malondialdehyde content in *Rosa chinensis* seedlings after drought stress. The small letters (such as 'a' and 'b') in the bar graphs of your figures typically indicate statistical significance among the treatments, which is often determined through post-hoc analysis after ANOVA testing, such as Tukey's test or LSD (Least Significant Difference) test.

The level of MDA is an important indicator of plant stress resistance. Experimental results (**Figure 6**) show that under dry stress, the MDA level in the leaf of *Rhododendron*, *Jasminum sambac* and *Rosa chinensis*, both inoculated and non-inoculated with *P. indica*, initially decreased and then showed an increasing trend. This pattern suggests that at the onset of drought stress, active cellular regulation and maintenance of internal nutrients may suppress lipid peroxidation, reducing MDA content. However, as stress duration extends, the intensification of lipid peroxidation disrupts this balance, leading to a significant increase in MDA content later on.

At the conclusion of the drought stress phase, the MDA content of *P. indica*-inoculated *Jasminum sambac* was reduced compared to non-inoculated counterparts, whereas the differences in *Rhododendron* and *Rosa chinensis* were negligible. The MDA content in *Rhododendron*, *Jasminum sambac*, and *Rosa chinensis* was respectively 6.8%, 10.4% and 7.9% lower than in the control. Across three times intervals, MDA levels in non-inoculated cuttings were consistently higher than those inoculated with *P. indica*, suggesting that *P. indica* inoculation may reduce MDA levels in plants, mitigating membrane damage under adverse conditions and improving drought resistance.

### 3.4 The influence of *P. indica* on chlorophyll content in cuttings under drought stress

**Figure 7:**
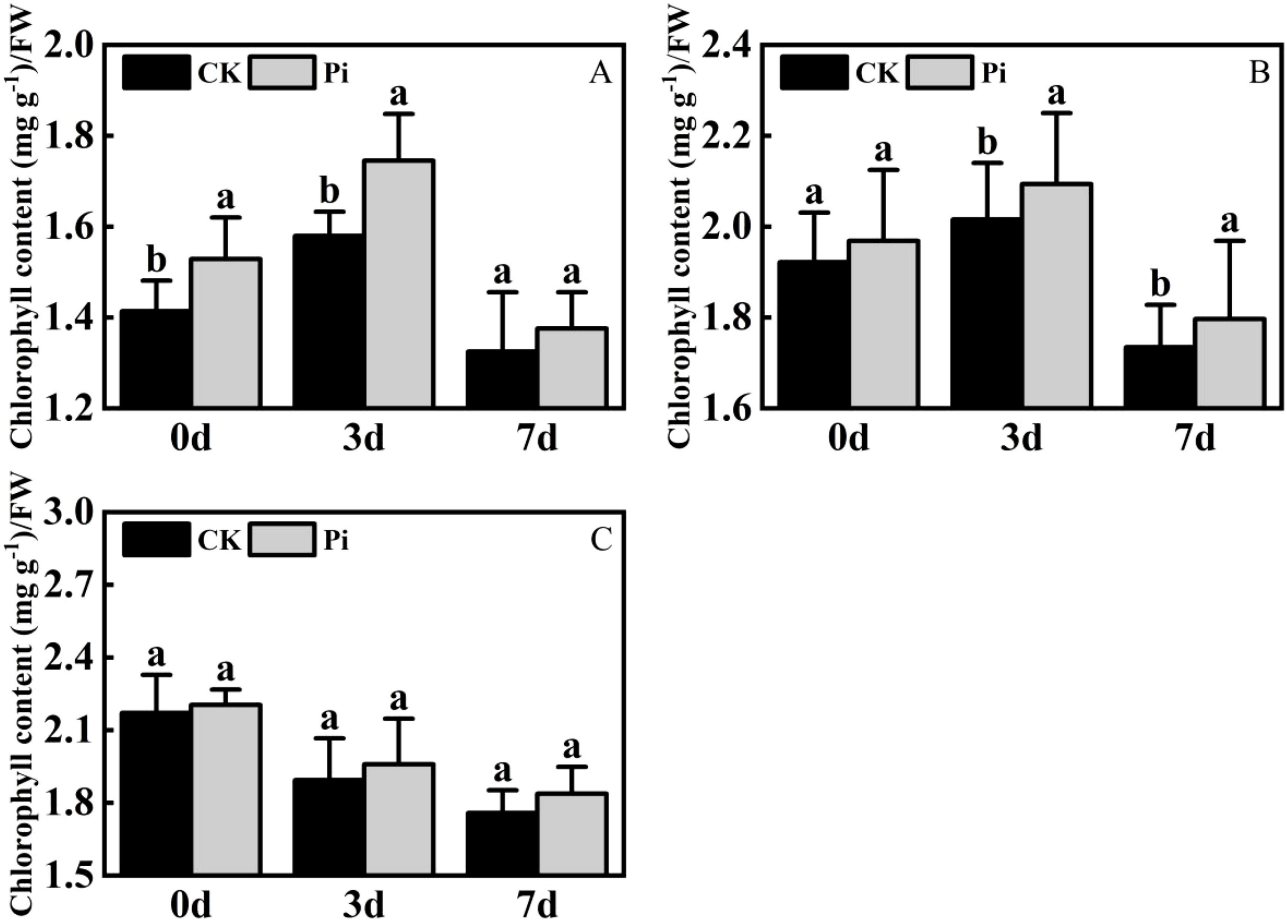
Chlorophyll content after drought stress. **(A)** Chlorophyll content in *Rhododendron* seedlings after drought stress: **(B)** Chlorophyll content in *Jasminum sambac* seedlings after drought stress: **(C)** Chlorophyll content in *Rosa chinensis* seedlings after drought stress. The small letters (such as 'a' and 'b') in the bar graphs of your figures typically indicate statistical significance among the treatments, which is often determined through post-hoc analysis after ANOVA testing, such as Tukey's test or LSD (Least Significant Difference) test.

Under dry conditions, the total chlorophyll level in *Rhododendron* and *Jasminum sambac* inoculated with *P. indica* exhibited an initial increase followed by a decrease, reaching a peak on the third day of drought treatment. In contrast, *Rosa chinensis* showed a continuous decline in total chlorophyll content from the onset of drought stress (**Figure 7**). During the initial stages of drought stress, the application of *Rhododendron* and *Jasminum sambac* cuttings has been demonstrated to enhance drought resistance by improving photosynthesis, resulting in an *a priori* increase in Chlorophyll content in non-inoculated and *P. indica*-inoculated leaves. This increase was observed to reach a peak on 3rd day. At this peak, the chlorophyll content in *P. indica*-inoculated *Rhododendron* leaves increased by 10.1% compared to the control, and *Jasminum sambac* increased by 8%, then gradually declined, indicating that *P. indica* inoculation helps to slow the decrease of chlorophyll in leaf cells. Chlorophyll content in *Rosa chinensis* leaves continuously decreased from the beginning. At all stages of drought stress, the chlorophyll levels in *P. indica*-inoculated leaves were found to be greater than in non-inoculated ones. Furthermore, by the end of the stress period, the chlorophyll content in *P. indica*-inoculated plants was found to be 10% higher compared to the control. The findings indicate that under drought stress, *P. indica*-inoculated cuttings may alleviate the effects of drought by increasing chlorophyll content to sustain photosynthesis, also marginally affecting the rate of decline in total chlorophyll content in plant leaves experiencing dehydration stress.

### 3.5 The effect of *P. indica* on 3,3’-diaminobenzidine and Nitroblue tetrazolium staining in cuttings under drought stress

**Figure 8:**
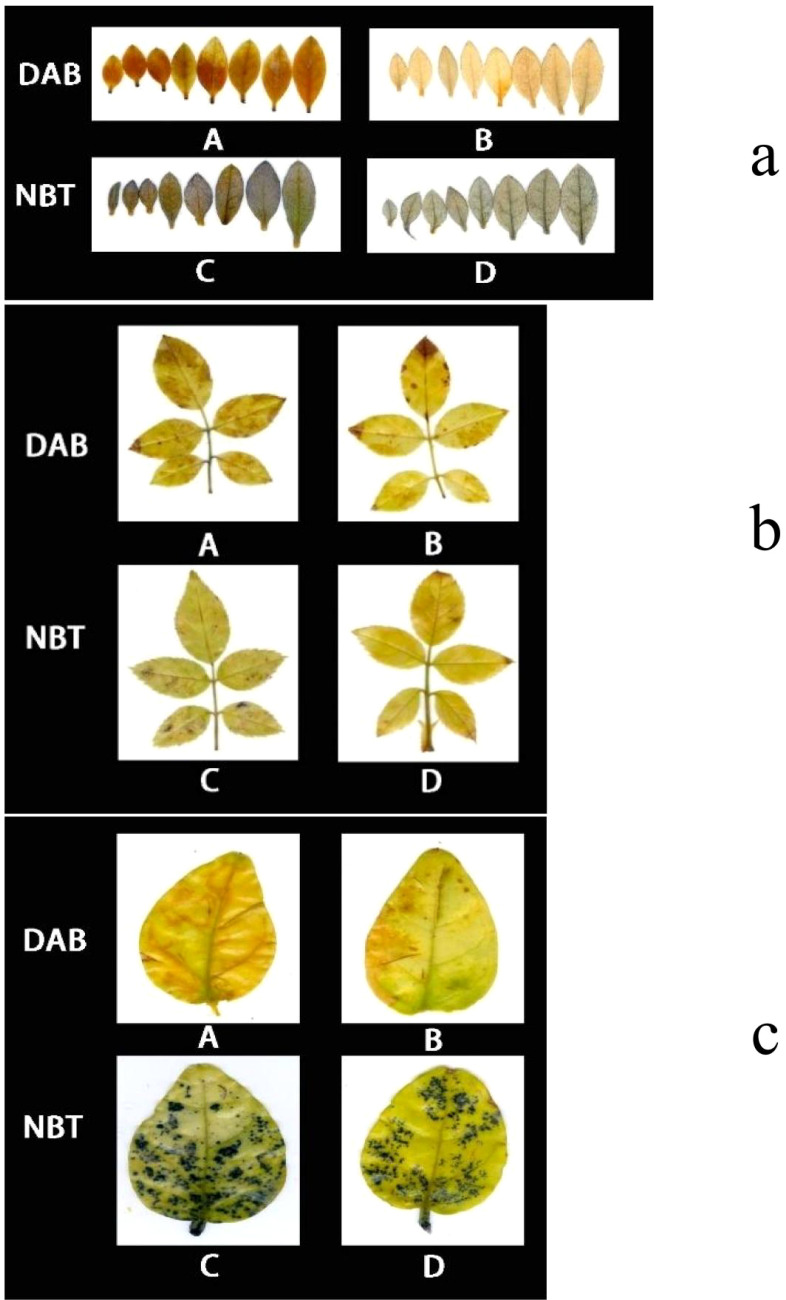
**3,3’-**Diaminobenzidine (DAB) and Nitroblue Tetrazolium (NBT) staining results of *Rhododendron*, *Rosa chinensis*, and *Jasminum sambac* cuttings. **(A)** DAB staining results of *Rhododendron* cuttings (a). *Rosa chinensis* cuttings (b), and *Jasminum sambac* cuttings (c). **(B)** DAB staining results of *Rhododendron* cuttings inoculated with *Piriformospora indica* (a), *Rosa chinensis* cuttings inoculated with *Piriformospora indica* (b), and *Jasminum sambac* cuttings inoculated with *Piriformospora indica* (c). **(C)** NBT staining results of *Rhododendron* cuttings (a), *Rosa chinensis* cuttings (b), and *Jasminum sambac* cuttings (c). **(D)** NBT staining results of *Rhododendron* cuttings inoculated with *Piriformospora indica* (a), *Rosa chinensis* cuttings inoculated with *Piriformospora indica* (b), and *Jasminum sambac* cuttings inoculated with *Piriformospora indica* (c).

As demonstrated in **Figure 8A**, following DAB (3,3’-Diaminobenzidine) decolorization, the leaves of *Rhododendron* inoculated with *Piriformospora indica* exhibited a yellowish-white coloration, whereas the leaves of non-inoculated plants predominantly displayed a brown hue. This observation suggests a lower accumulation of hydrogen peroxide (H_2_O_2_) in the inoculated plants compared to the control group. Similarly, the NBT (Nitroblue tetrazolium) staining results presented in **Figure 8** indicated that while both inoculated and non-inoculated plants showed blue staining, the intensity of blue in non-inoculated plants was considerably darker, suggesting elevated levels of superoxide anions (O_2_^−^). These findings imply enhanced reactive oxygen species (ROS) scavenging capacity in *P. indica*-inoculated plants. This is further supported by the enzymatic activity data presented in **Table 3**, where a significant increase in superoxide dismutase (SOD) activity (251.23 U g^−^¹ in inoculated vs. 237.58 U g^−^¹ in control) and peroxidase (POD) activity (119.65 U g^−^¹ in inoculated vs. 105.85 U g^−^¹ in control) was observed in the inoculated *Rhododendron* plants.

**Table 3:** SOD and POD contents in leaves.

	U g^-1^	Rhododendron	Jasminum sambac	Rosa chinensis
SOD	CK	237.58 ± 11.99b	72.48 ± 14.58b	109.54 ± 16.48a
	Pi	251.23 ± 18.29a	79.73 ± 6.73a	118.35 ± 9.80a
POD	CK	105.85 ± 8.15b	13.36 ± 2.49b	104.65 ± 12.56b
	Pi	119.65 ± 9.57a	16.82 ± 2.84a	112.29 ± 13.45a

For *Rosa chinensis* (**Figure 8B**), DAB staining showed brown spots in both inoculated and non-inoculated leaves. However, the spots in the inoculated group were smaller, indicating that the oxidative stress level of plants treated with *P. indica* was reduced. NBT staining did not show significant differences in the blue area between the two groups, suggesting that the activity of SOD in rose may not be so obvious under the tested conditions. Nevertheless, enzymatic assays (**Table 3**) showed that after inoculation with *P. indica*, the SOD activity in rose (118.35 U g-1 in the inoculated group and 109.54 U g-1 in the control group) did not increase significantly, while the POD activity (112.29 U g-1 in the inoculated group and 104.65 U g-1 in the control group) increased significantly, confirming the reduction of oxidative damage.

In the case of *Jasminum sambac* (**Figure 8C**), DAB staining also revealed brown patches in both inoculated and non-inoculated plants. However, the inoculated plants exhibited smaller brown areas, indicating lower H_2_O_2_ accumulation in comparison to the non-inoculated group. NBT staining revealed widespread blue spots in both groups, with the non-inoculated plants displaying slightly larger blue areas, which suggests higher levels of O_2_^−^. Corresponding to these observations, the enzyme activity analysis (**Table 3**) demonstrated significantly higher SOD activity (79.73 U g^−^¹ in inoculated vs. 72.48 U g^−^¹ in control) and POD activity (16.82 U g^−^¹ in inoculated vs. 13.36 U g^−^¹ in control) in *Jasminum sambac*, further supporting the hypothesis that *P. indica* enhances antioxidant activity.

In summary, across all three plant species—*Rhododendron*, *Rosa chinensis*, and *Jasminum sambac*—the combined results from DAB and NBT staining, together with the enzymatic activity data, suggest that *P. indica* inoculation significantly enhances the antioxidant defense system. The increased SOD and POD activities observed in inoculated plants indicate a more efficient ROS scavenging mechanism, leading to reduced oxidative stress under drought conditions. These findings provide compelling evidence that *P. indica* plays a crucial role in improving the oxidative stress tolerance of these species, as reflected by the lighter staining and higher antioxidant enzyme activity in the inoculated plants.

### 3.6 The impact of *P. indica* on chlorophyll fluorescence in cuttings under high-temperature stress

**Figure 9:**
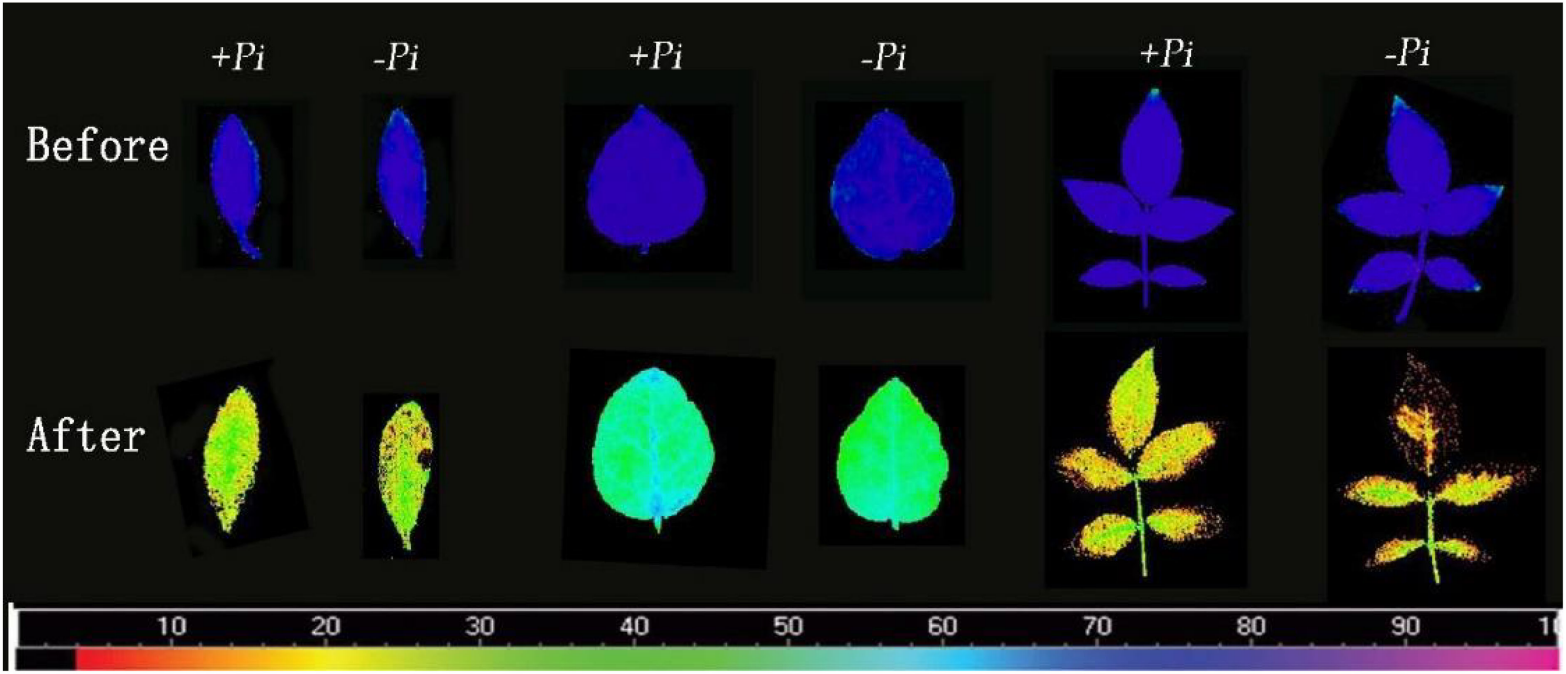
Fv/Fm chlorophyll fluorescence images of cutting seedlings before and after high temperature stress. The first two are rhododendron leaves, the middle two are moon leaves, and the last two are *Jasminum sambac* leaves.The color scale beneath the figures ranges from 0 (black) to 100 (pink), indicating the degree of leaf damage from high to low.

**Figure 10:**
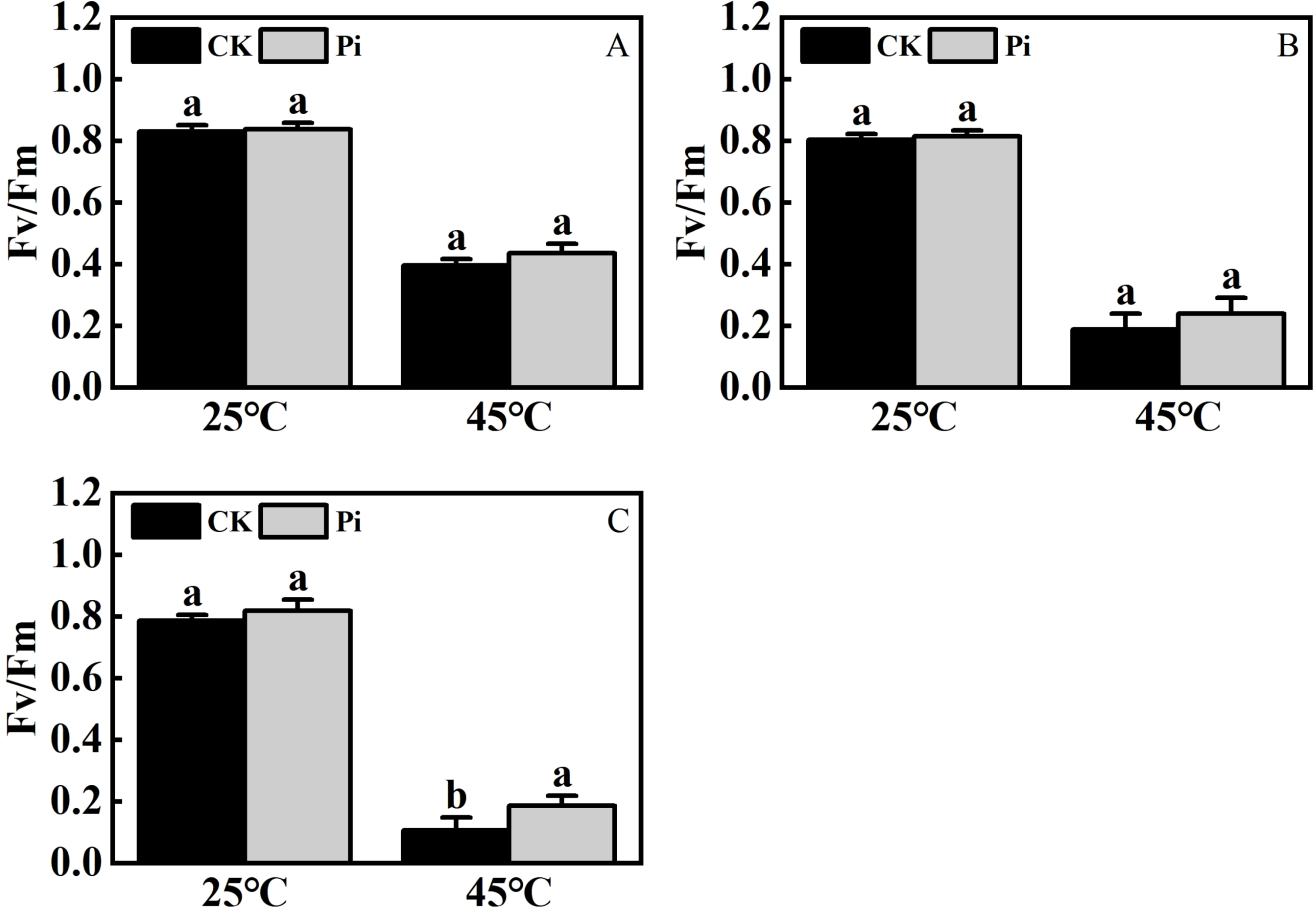
The effect of high temperature stress on FV/Fm cutting seedling. **(A)** FV/Fm ratio of *Rhododendron* cuttings after heat stress: **(B)** FV/Fm ratio of *Jasminum sambac* cuttings after heat stress: **(C)** FV/Fm ratio of *Rosa chinensis* cuttings after heat stress. The small letters (such as 'a' and 'b') in the bar graphs of your figures typically indicate statistical significance among the treatments, which is often determined through post-hoc analysis after ANOVA testing, such as Tukey's test or LSD (Least Significant Difference) test.

Research on the impact of *P. indica* on the photosynthetic system of plants under heat stress, particularly the response of the chlorophyll fluorescence system to high temperatures and its heat tolerance mechanisms, remains limited. This study examines the impact of *P. indica* on the chlorophyll fluorescence characteristics of cuttings under heat stress, starting from the dynamics of chlorophyll fluorescence. As shown in **Figure 9**, after exposure to 45°C heat stress, changes of varying degrees were observed in the Fv/Fm images of *Rhododendron*, *Jasminum sambac*, and *Rosa chinensis* leaves compared to the control. After high-temperature treatment, all leaves shifted away from the blue region, with *Rosa chinensis* experiencing the most severe heat damage. Both control and *P. indica*-inoculated *Rosa chinensis* leaves showed various degrees of scorching, although the control leaves were more severely scorched. *Rhododendron* also showed some scorching in both groups, whereas *Jasminum sambac* leaves, both control and *P. indica*-inoculated, did not scorch but exhibited color changes. High temperatures can affect the electron transfer process of PSII, altering the rate of photosynthesis. PSII is heat-sensitive, and its activity is severely inhibited by high temperatures. **Figure 10** demonstrate that after 24 hours of 45°C heat stress, the decline in Fv/Fm in *P. indica*-inoculated cuttings was less than in non-inoculated ones. Post heat stress, the Fv/Fm of *Rhododendron*, *Jasminum sambac*, and *Rosa chinensis* leaves all decreased, improving by 27.3%, 10.3%, and 51.1% compared to the control, respectively, with *Rosa chinensis* leaves showing a significant difference. These results suggest that *P. indica* may enhance the chloroplasts’ heat tolerance in cuttings under heat stress to some extent.

### 3.7 The effect of *P. indica* on rhododendron tissue-cultured seedlings under high-temperature stress

**Figure 11:**
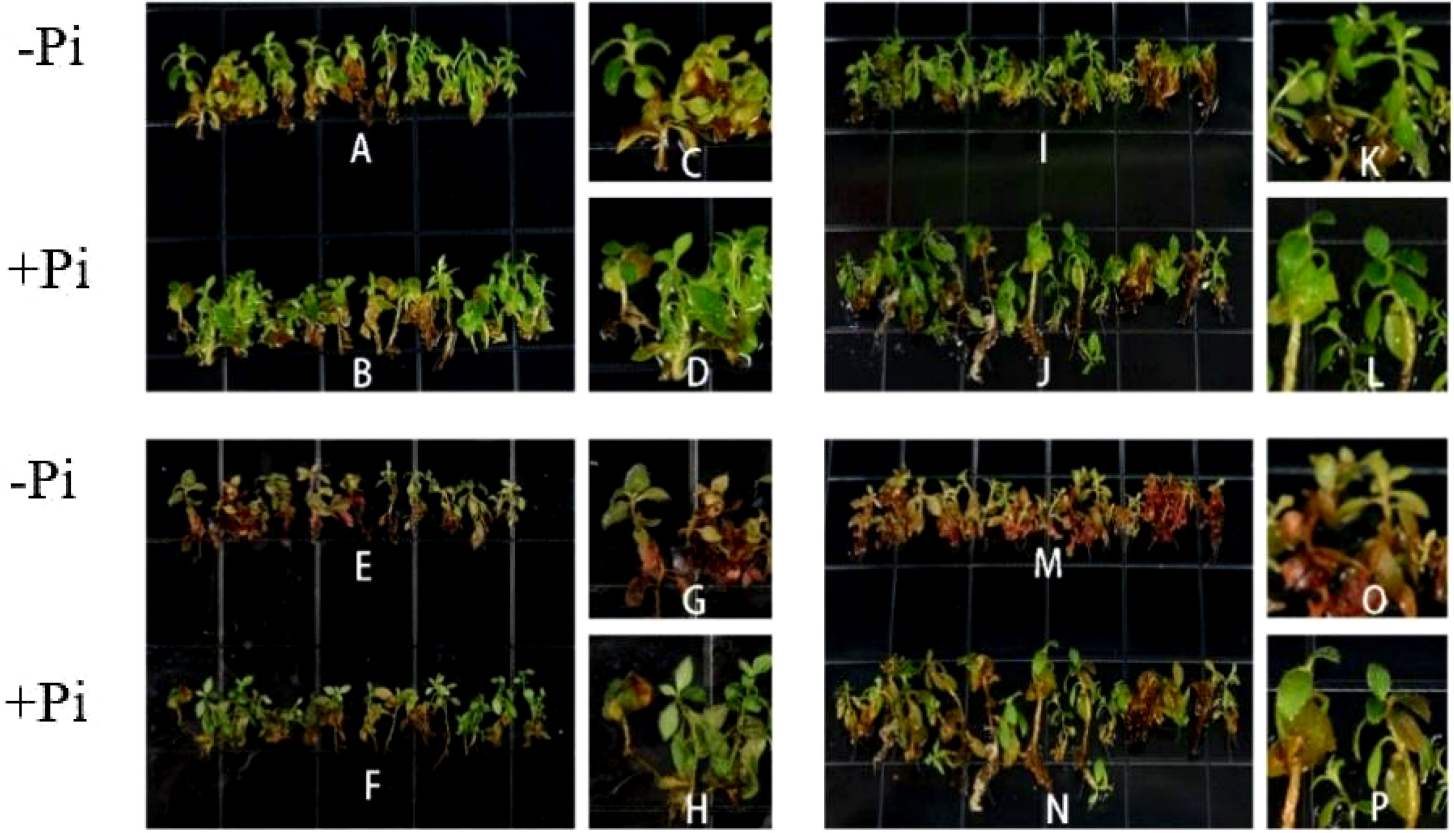
Tissue Culture seedlings of *Rhododendron* under high temperature stress. **(A, I)** Tissue cultured seedlings of *Rhododendron* before high temperature stress: **(B, J)** Tissue cultured seedlings of *Rhododendron* inoculated with *P. indica* before high temperature stress: **(E, M)** Tissue cultured seedlings of Rhododendron after high temperature stress: **(F, N)** Tissue cultured seedlings of Rhododendron inoculated with *P. indica* after high temperature stress: **(C, D, K, L)** Detail picture before high temperature stress: **(G, H, O, P)** Detail picture after high temperature stress.

**Figure 12:**
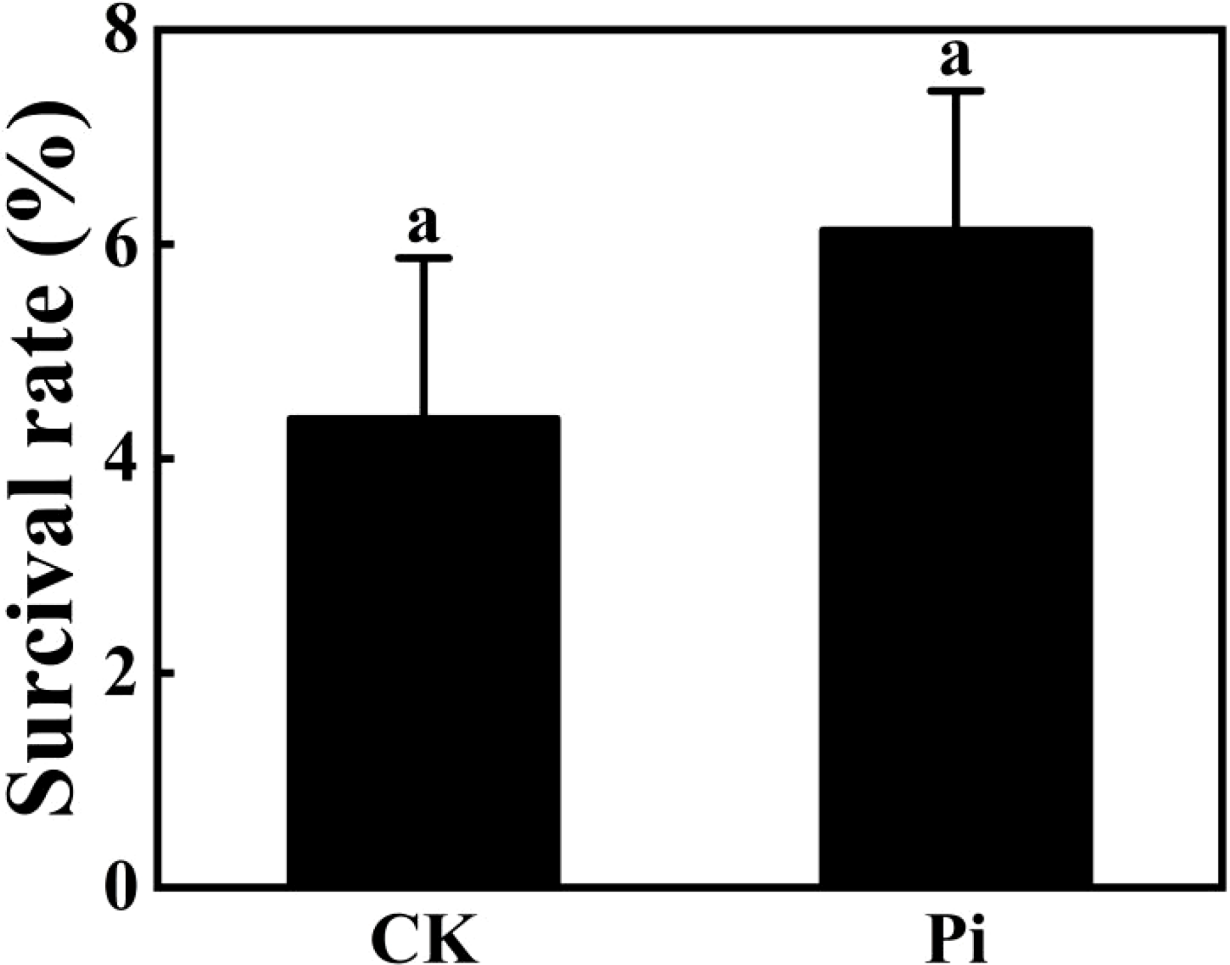
Survival rate of *Rhododendron* plantlets inoculated and uninoculated with *Piriformospora indica* at 42°C. The small letters (such as 'a' and 'b') in the bar graphs of your figures typically indicate statistical significance among the treatments, which is often determined through post-hoc analysis after ANOVA testing, such as Tukey's test or LSD (Least Significant Difference) test.

The preliminary selection temperature for high-temperature screening was set at 42°C. Hence, *Rhododendron* tissue-cultured seedlings, both inoculated and non-inoculated with *P. indica*, were subjected to this temperature for 24 hours (**Figure 11**). Post-treatment observations revealed some variations in survival rates (**Figure 12**), with the control group comprising 25 seedlings, of which only one survived and 24 wilted, resulting in an approximate survival rate of 4%. The *P. indica*-inoculated group, consisting of 29 seedlings, had two survivors and 27 wilted, with a survival rate of about 6%. These results indicate a slight improvement in high-temperature tolerance in *P. indica*-inoculated tissue-cultured seedlings compared to the control, although the increase was not as significant as anticipated.

**Figure 13:**
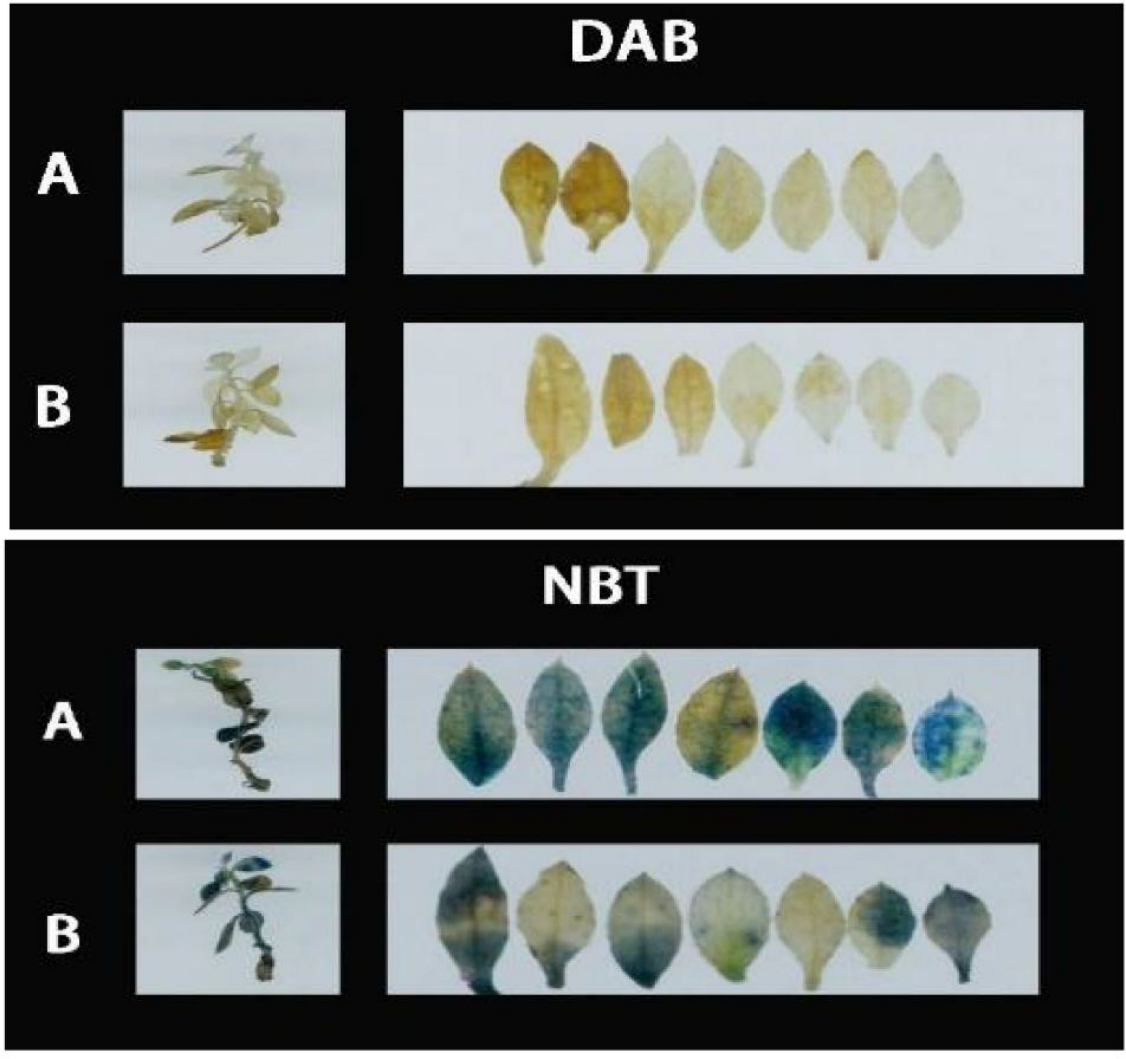
DAB (3,3’-Diaminobenzidine) and Nitroblue Tetrazolium (NBT) staining results of *Rhododendron* tissue culture seedlings. **(A)** DAB staining results showing the presence of reactive oxygen species (ROS) in *Rhododendron* tissue culture seedlings without *Piriformospora indica* inoculation (top panel) and with *Piriformospora indica* inoculation (bottom panel). **(B)** NBT staining results indicating the accumulation of superoxide in *Rhododendron* tissue culture seedlings without *Piriformospora indica* inoculation (top panel) and with *Piriformospora indica* inoculation (bottom panel).

After DAB Decolorization (**Figure 13**), it was observed that *Rhododendron* leaves, both inoculated and non-inoculated with *P. indica*, developed brown patches, with the non-inoculated leaves exhibiting a deeper shade of brown. According to the NBT staining results (**Figure 13**), both groups displayed significant blue areas, albeit those on non-inoculated leaves were larger and of a darker hue. The outcomes of both staining methods suggest that following high-temperature stress, *P. indica* may play a role in enhancing the activity of SOD and POD enzymes in *Rhododendron*.

## 4 Discussion

### 4.1 The impact of *P. indica* on the wilting severity of cuttings under drought stress

This study found that inoculation with *P. indica* significantly alleviated wilting severity in cuttings subjected to drought stress. Observations of *Rhododendron*, *Jasminum sambac*, and *Rosa chinensis* cuttings demonstrated that inoculated plants exhibited a marked reduction in wilting severity compared to the control group, with wilting rates decreasing by 13%, 17%, and 16.6%, respectively, following drought stress.Several potential mechanisms have been proposed to explain this phenomenon. One hypothesis suggests that *P. indica* enhances plant drought resistance by promoting root development and increasing water absorption capacity. Previous studies have reported that *P. indica* forms symbiotic relationships with various crops, including rice, wheat, barley, and sorghum, promoting both vegetative and reproductive growth. Additionally Vahabi et al. (2018) demonstrated that P. indica can facilitate systemic jasmonate responses across plants through its hyphal network, which may enhance plant hormone signaling and contribute to improved stress resistance. These effects include increases in root length, root volume, fresh root mass, fresh stem mass, number of leaves, leaf area, plant height, and biomass yield (Ghaffari et al., 2019; Hosseini et al., 2018; Saddique et al., 2018; Xu et al., 2017). Another hypothesis posits that *P. indica* activates endogenous drought resistance signaling pathways within the plant, enhancing the accumulation of osmotic regulatory substances, such as proline and sugars. These compounds help the plant maintain cellular osmotic balance, mitigating the impact of drought stress (Swetha and Padmavathi, 2020). For example, Saddique et al. (2018) demonstrated that colonization by *P. indica* upregulated the activity of Δ¹-pyrroline-5-carboxylate synthase (P5CS), a key enzyme in proline synthesis, thereby increasing proline levels and enhancing the overall antioxidant capacity of leaves. Furthermore, other studies have reported that *P. indica* inoculation promotes the production of antioxidant enzymes, such as superoxide dismutase (SOD) and peroxidase (POD), which help protect plants from oxidative stress caused by drought (Cao et al., 2023; Xu et al., 2017). These mechanisms collectively enable cuttings to maintain normal physiological functions under drought conditions, thereby reducing the severity of wilting and improving drought tolerance.

### 4.2 The effect of *P. indica* on MDA content, SOD, and POD activities in cuttings under drought stress

Inoculation with *P. indica* can enhance the activities of SOD and POD within the plant, thereby reducing the plant’s internal MDA content (Cao et al., 2023). Experimental results have shown that the MDA content in Jasminum sambac, Rhododendron, and Rosa chinensis leaves subjected to drought stress and not inoculated was significantly lower than in the CK. Xu et al. (2022) reported that the coordination of root auxin with P. indica and Bacillus cereus significantly enhanced root rhizosheath formation under soil drying conditions, which likely contributes to the plant's ability to tolerate drought stress by improving water absorption and root functionality. Staining results also indicated significant differences in SOD and POD activities between the two treatments (**Table 3**). MDA is the final product of lipid peroxidation, and an increase in its content is an important indicator of cell membrane damage. Current research offers several possible explanations for this phenomenon. Firstly, inoculation with *P. indica* might enhance antioxidant enzyme activities (such as superoxide dismutase and catalase). For example, tsai and colleagues discovered that colonization by *P. indica* promoted increased activities of catalase and glutathione reductase, raising the ratio of reduced to oxidized glutathione and reducing the internal MDA content of the plants, though it had no significant effect on the activity of superoxide dismutase (Tsai et al., 2020). Furthermore, research by Hosseini and others found that *P. indica* colonization significantly increased root length, root volume, leaf water potential, relative water content of leaves, and chlorophyll content in wheat. The activities of catalase and ascorbate peroxidase were significantly reduced (Ghaffari et al., 2019), potentially because *P. indica* modulates stress-induced oxidative stress, inhibiting the formation and excessive accumulation of reactive oxygen species in plant cells, thus enhancing the crop’s stress resistance (Kheyri et al., 2022). This indicates that *P. indica* can effectively mitigate oxidative stress caused by drought stress, maintaining cell membrane integrity, and thereby improving plant drought resistance (Dabral et al., 2019). However, the mechanisms involved require further investigation.

### 4.3 The effect of *P. indica* on chlorophyll content in cuttings under drought stress

This investigation revealed that inoculation with *P. indica* mitigates the reduction of chlorophyll content in cuttings subjected to drought stress. Previous studies have demonstrated that *P. indica* colonization enhances the vegetative growth of various crops, such as wheat, cucumber, and rice, under drought conditions. This improvement results in an increase in chlorophyll content and photosynthetic parameters, delays the decline in photosynthetic efficiency, and mitigates the degradation of chlorophyll and thylakoid proteins, thereby reducing photodamage (Atia et al., 2020; Zhang et al., 2022). Research by Tsai et al. (2020) found that *P. indica* inoculation elevated chlorophyll levels in rice, promoting stomatal closure, increasing leaf temperature, enhancing Fv/Fm, and reducing the extent of leaf wilting and damage to photosynthetic efficiency. The underlying mechanisms may involve the symbiotic effects of *P. indica*, which enhance the expression of drought-responsive genes such as DREB2A, CBL1, ANAC072, RD29A, and microRNAs miR159 and miR396. This upregulation strengthens mRNA levels and increases the protein content of Ca²^+^-sensing regulators on the thylakoid membrane, promoting the accumulation of photosynthesis-related proteins and, consequently, boosting chlorophyll content (Ghaffari et al., 2019; Mohsenifard et al., 2017). In summary, *P. indica* inoculation can alleviate the decline in chlorophyll content in cuttings under drought stress, potentially by modulating drought-responsive genes and enhancing Ca²^+^ regulation to increase the levels of photosynthesis-related proteins, consistent with prior research findings.

### 4.4 The effect of *P. indica* on chlorophyll fluorescence in cuttings under high-temperature stress

**Figure 14:**
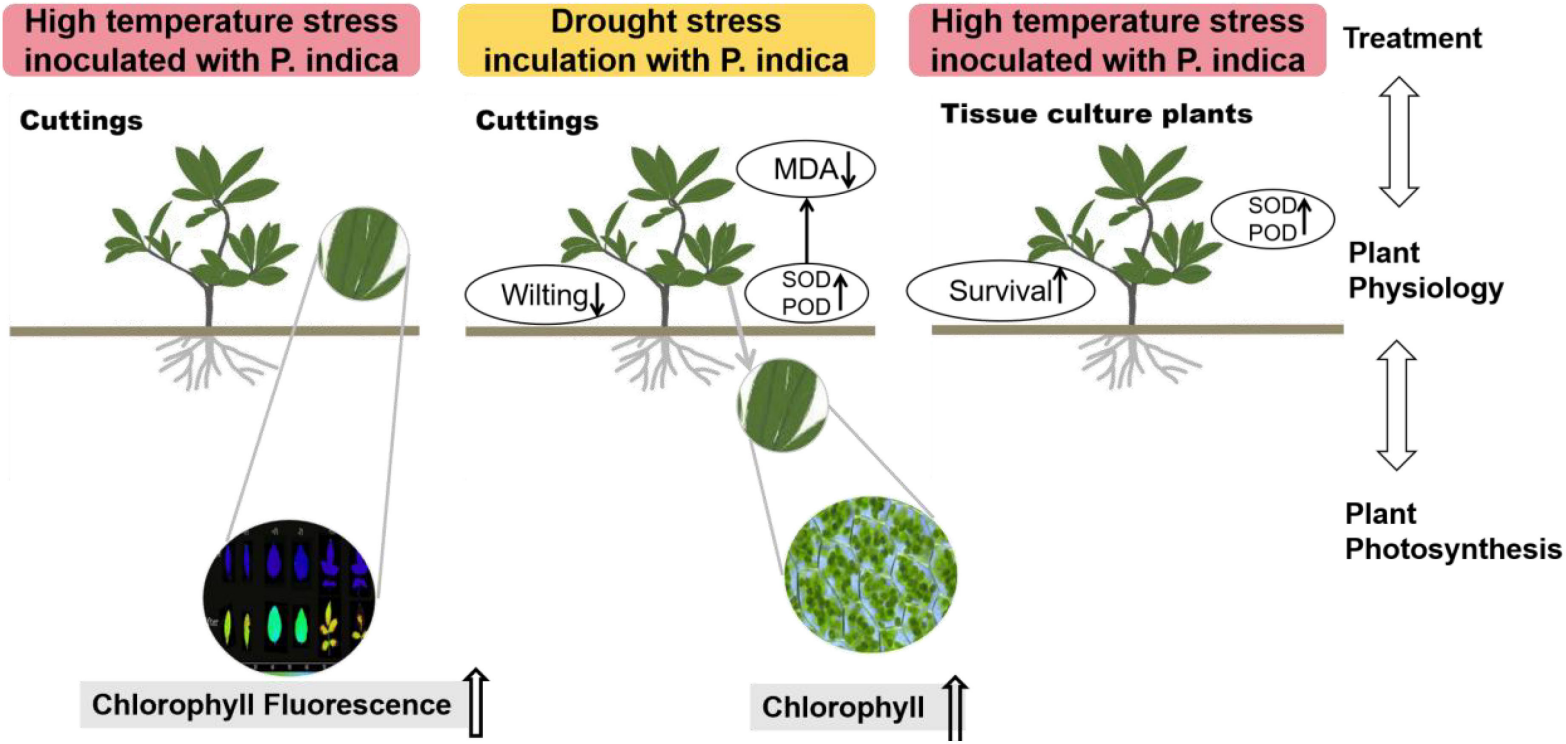
Effects on the physiological characteristics of plants inoculated with *Piriformospora indica* under high temperature and drought stress conditions.

The results of this study underscore the potential role of *P. indica* in enhancing the heat stress tolerance of cuttings, particularly by fortifying the heat resistance of chloroplasts. As shown in **Figure 14**, This finding aligns with previous research, though the precise mechanisms require further exploration. Studies suggest that *P. indica* inoculation alleviates physiological stress induced by high temperatures by optimizing water utilization efficiency and enhancing cellular water retention capacity (Bandyopadhyay et al., 2022). Shahabivand et al. (2017) also demonstrated that *P. indica* positively influenced chlorophyll fluorescence and growth in sunflower under cadmium toxicity, indicating that the endophytic fungus may have a broader role in mitigating various types of environmental stress. This effect may be achieved by promoting the accumulation of osmotic regulatory substances, such as proline, which help maintain intracellular water balance and mitigate adverse effects on photosynthesis. It also sustains high levels of chlorophyll fluorescence parameters such as Fv/Fm, ensuring photosynthetic efficiency (Liu et al., 2022). Moreover, research has shown that under high-temperature stress, plants accumulate significant amounts of reactive oxygen species (ROS), which can damage the photosynthetic system, particularly PSII, thereby impairing chlorophyll fluorescence characteristics (Kimm et al., 2021). *P. indica* strengthens the plant’s antioxidative defense system by increasing the activity of key antioxidative enzymes, including superoxide dismutase (SOD), peroxidase (POD), and catalase (CAT). These enzymes help remove excessive ROS, protect the photosynthetic apparatus, and maintain optimal chlorophyll fluorescence performance (Abdelaziz et al., 2019, 2021). Further studies also suggest that *P. indica* modulates plant hormone levels, such as gibberellin (GA), indole-3-acetic acid (IAA), and abscisic acid (ABA), influencing plant growth and stress response mechanisms, particularly under high-temperature conditions. For example, increased ABA levels during stress responses can reduce water loss, indirectly supporting chlorophyll fluorescence and maintaining photosynthetic efficiency (An et al., 2023). In summary, the multifaceted role of *P. indica* in plant responses to heat stress highlights its potential application and lays a crucial theoretical foundation for future research.

### 4.5 The effect of *P. indica* on rhododendron tissue-cultured seedlings under high-temperature stress

The results of this study underscore the potential role of *P. indica* in enhancing the heat stress tolerance of cuttings, particularly by fortifying the heat resistance of chloroplasts. This finding aligns with previous research, though the specific mechanisms require further exploration. Studies suggest that *P. indica* inoculation alleviates physiological stress induced by high temperatures by optimizing water use efficiency and enhancing cellular water retention capacity (Bandyopadhyay et al., 2022). This effect may be achieved by promoting the accumulation of osmotic regulation substances, such as proline, which aid in maintaining intracellular water balance, mitigating the adverse effects on photosynthesis, and sustaining high levels of chlorophyll fluorescence parameters such as Fv/Fm (Liu et al., 2022). Furthermore, research has shown that high-temperature stress triggers the accumulation of reactive oxygen species (ROS) in plants, which can damage the photosynthetic system, particularly PSII, thus impairing chlorophyll fluorescence characteristics (Kimm et al., 2021). In response, *P. indica* enhances the plant’s antioxidative defense system by increasing the activity of enzymes like superoxide dismutase (SOD), peroxidase (POD), and catalase (CAT). These enzymes help clear excessive ROS, protect the photosynthetic apparatus, and sustain optimal chlorophyll fluorescence performance (Abdelaziz et al., 2019, 2021). Additionally, studies indicate that *P. indica* may modulate plant hormone levels, such as gibberellin (GA), indole-3-acetic acid (IAA), and abscisic acid (ABA), influencing plant growth and stress response mechanisms, especially under high-temperature conditions. For example, increased ABA levels during stress responses can reduce water evaporation, indirectly maintaining chlorophyll fluorescence parameters and protecting photosynthetic efficiency (An et al., 2023). In summary, the multifaceted role of *P. indica* in plant responses to heat stress not only highlights its potential application but also provides an important theoretical foundation and direction for future research.

### 4.6 Limitations of the study

While this study has provided valuable insights, there are several limitations that should be acknowledged. Firstly, we did not employ specific quantitative methods such as measuring relative water content (RWC), assessing ion leakage for membrane integ rity, or conducting cell viability staining with Trypan blue. Instead, our findings were predominantly based on phenotypic observations and survival rates determined through visual inspection. Although this approach allowed for an initial assessment of the effects, it lacks the precision of more rigorous quantitative methods, potentially limiting the depth and accuracy of our conclusions regarding the physiological responses to the treatments.

Secondly, leaf size was not systematically measured during the experiment. Since our study primarily focused on phenotypic characteristics following staining, leaf size data were regarded as a secondary factor, leading to inconsistencies and a lack of precise measurements. Additionally, the absence of a scale bar in the images further emphasizes this limitation, as such details could have provided more context to the visual data presented.

## 5 Conclusion

The present study focused on *Jasminum sambac*, *Rosa chinensis* cuttings, and *Rhododendron* tissue-cultured seedlings, subjecting them to drought and high-temperature stress experiments with the objective of exploring the regulatory mechanisms of *P. indica* on plants under drought and high-temperature conditions. The findings include the following: (1) In the context of drought stress, it can be posited that *P. indica* may act to inhibit the rise in MDA content within the cuttings, while also mitigating the loss of chlorophyll content and reducing photosynthetic loss. In the context of high-temperature stress, *P. indica* may mitigate the damage to leaves by enhancing the heat resistance of chloroplasts in the cuttings. (2) The results of the DAB and NBT staining of the antioxidative enzyme activities demonstrated color differences among the *Rhododendron*, *Jasminum sambac*, and *Rosa chinensis* staining groups, which indirectly indicated that *P. indica* had an impact on the plant’s internal SOD and POD antioxidative enzyme content. In conclusion, the inoculation of *P. indica* has been demonstrated to enhance the resistance of *Rhododendron, Jasminum sambac*, and *Rosa chinensis* to drought and high-temperature conditions.

## Data Availability

The original contributions presented in the study are included in the article/supplementary material. Further inquiries can be directed to the corresponding authors.
